# Views and experiences of healthcare professionals and patients on the implementation of a 23-hour accelerated enhanced recovery programme: a mixed-method study

**DOI:** 10.1186/s12913-024-10837-z

**Published:** 2024-03-13

**Authors:** Misha A. T. Sier, Eva Godina, Thaís T. T. Tweed, Imane Daher, Jan H. M. B. Stoot

**Affiliations:** 1 Department of Surgery, Zuyderland Medical Centre, Henri Dunantstraat 5, Heerlen, 6419 PC The Netherlands; 2 School of Nutrition and Translational Research in Metabolism (NUTRIM), Maastricht University, Universiteitssingel 40, Maastricht, 6229 ER The Netherlands; 3 Faculty of Health, Medicine and Life Sciences (FHML), Maastricht University, Universiteitssingel 40, Maastricht, 6229 ER The Netherlands; 4 Department of Surgery, Maastricht University Medical Centre+, P. Debyelaan 25, Maastricht, 6229 HX the Netherlands; 5 Department of Gastroenterology, Zuyderland Medical Centre, Henri Dunantstraat 5, Heerlen, 6419 PC the Netherlands

**Keywords:** Colorectal cancer surgery, Patient perspective, Health care professional perspective, Accelerated recovery, Perioperative cancer care

## Abstract

**Background:**

An accumulating body of research suggests that an accelerating enhanced recovery after colon surgery protocol is beneficial for patients, however, to obtain these effects, adherence to all elements of the protocol is important. The implementation of complex interventions, such as the Enhanced Recovery After Surgery protocol (ERAS), and their strict adherence have proven to be difficult. The same challenges can be expected in the implementation of the accelerated Enhanced Recovery Pathways (ERPs). This study aimed to understand the perspectives of both healthcare professionals (HCPs) and patients on the locally studied acCelerated enHanced recovery After SurgEry (CHASE) protocol.

**Methods:**

For this mixed-method study, HCPs who provided CHASE care and patients who received CHASE care were recruited using purposive sampling. Ethical approval was obtained by the Medical Ethical Committee of the Zuyderland Medical Centre (NL71804.096.19, METCZ20190130, October 2022). Semi-structured, in-depth, one-on-one interviews were conducted with HCPs (*n* = 13) and patients (*n* = 11). The interviews consisted of a qualitative and quantitative part, the protocol evaluation and the Measurement Instrument or Determinant of Innovations-structured questionnaire. We explored the perspectives, barriers, and facilitators of the CHASE protocol implementation. The interviews were audiotaped, transcribed verbatim and analysed independently by two researchers using direct content analysis.

**Results:**

The results showed that overall, HCPs support the implementation of the CHASE protocol. The enablers were easy access to the protocol, the relevance of the intervention, and thorough patient education. Some of the reported barriers included the difficulty of recognizing CHASE patients, the need for regular feedback, and the updates on the implementation progress. Most patients were enthusiastic about early discharge after surgery and expressed satisfaction with the care they received. On the other hand, the patients sometimes received different information from different HCPs, considered the information to be too extensive and few experienced some discomfort with CHASE care.

**Conclusion:**

Bringing CHASE care into practice was challenging and required adaptation from HCPs. The experiences of HCPs showed that the protocol can be improved further, and the mostly positive experiences of patients are a motivation for this improvement. These results yielded practical implications to improve the implementation of accelerated ERPs.

**Supplementary Information:**

The online version contains supplementary material available at 10.1186/s12913-024-10837-z.

## Introduction

In the last two decades, the Enhanced Recovery After Surgery (ERAS) program has been globally adopted as a standard of care for patients undergoing elective colorectal surgery. ERAS is a perioperative care protocol encompassing several components aimed at optimizing perioperative management, to reduce perioperative complications and enhance postoperative recovery [1–3]. The effectiveness of ERAS has been shown to depend on strict adherence to the protocol [4]. However, the evidence suggests that adherence to all facets of ERAS can be challenging and that the adoption of programs like ERAS can be slow. Studies emphasize the importance of understanding both the barriers and enablers in the implementation of complex interventions [5]. After experiencing the successes of ERAS and its implementation, clinicians gained interest in further optimizing perioperative care. An expanding body of evidence indicates the feasibility and positive impact of accelerated Enhanced Recovery Pathways (ERPs), including the reduction of length of hospital stay (LOS) without adversely affecting postoperative outcomes. These accelerated ERPs have the potential to become the new standard treatment [6–11]. Data regarding the implementation of accelerated ERPs is limited, whereas, for the implementation of ERAS, several studies have been conducted to identify enablers and barriers.

Qualitative studies and implementation research have demonstrated that collaboration of involved stakeholders, leadership, profound education for healthcare professionals (HCPs) and patients, feedback on the implementation process, and outcome data facilitated the implementation of the ERAS protocol. Barriers included competing priorities, limited resources, changing behaviour of involved HCPs, limited personalized care, use of multiple guidelines, and visibility of ERAS [12–18].

The acCelerated 23-Hour stAy protocol after colon SurgEry (CHASE) is an accelerated ERP studied at the Zuyderland Medical Centre (ZMC). The CHASE protocol was inspired by the accelerated ERP of Levy and colleagues and was adjusted to align with the Dutch healthcare system [19]. The protocol was tested in the CHASE study [8]. In the CHASE protocol, certain pre-, peri- and postoperative ERAS elements are adjusted, such as analgesia, to further enhance recovery after surgery. The study demonstrated promising results, with 80% of the CHASE patients being discharged on postoperative day one without an increase in postoperative complications, and with high patient satisfaction [8].

To ensure the successful implementation of this accelerated ERP, understanding the facilitating factors and challenges for the implementation is key. The HCPs at the ZMC were already familiar with the ERAS protocol. However, it was anticipated that the barriers and facilitators would differ from ERAS implementation, due to the transition from ERAS to accelerated ERPs, which requires distinct education for patients and their relatives [20]. This study aimed to assess the views and experiences of HCPs and patients concerning the 23-h accelerated ERP.

## Methods

### Setting and study design

This mixed-method study was conducted at the ZMC. The choice of a mixed-method design aimed to collect qualitative data on the perspectives of HCPs and patients and quantitative data on established implementation determinants. Between 2020 and 2022, the accelerated ERP CHASE was studied for patients undergoing elective colon surgery at the ZMC, a 980-bed teaching hospital in the South of the Netherlands. The hospital employs 11 gastrointestinal surgeons, and during this timeframe, 165 surgeries were performed for malignant colon carcinoma. Specific pre-, peri- and postoperative ERAS elements are adjusted in the CHASE protocol to enable accelerated recovery and discharge on postoperative day one if patients meet the discharge criteria. The three key elements are optimal pain regimen (1), restricted fluid infusion (2) and truly minimally invasive surgery (3). The unique elements of the CHASE protocol are displayed below. Based on the positive results of the CHASE study, the incentive arose to implement CHASE for the studied patient population; patients classified as ASA I-II, aged 18–85 years, undergoing elective colon cancer surgery. During the CHASE implementation, ongoing data collection of patient outcomes was maintained.

Ethical approval was obtained by the Medical Ethical Committee of the Zuyderland Medical Centre (METC Z) (NL71804.096.19, METCZ20190130). The guidelines for Good Reporting of A Mixed Methods Study (GRAMMS) were adhered to [21], as well as standards for reporting qualitative research (SRQR) and the consolidated criteria for reporting qualitative research (COREQ) to enhance the quality of reporting in qualitative interview studies [22, 23].

### Research team

The interviews were conducted by the first author, a female Ph.D. student (M.S.) with prior experience in qualitative interviewing, under the guidance of a female experienced qualitative researcher (S.B.). The researchers conducting the interviews (M.S.) or performing the data analyses (M.S.) and E.G., a female medical student, both did not participate in patient care.

### Study population

For the interviews, the study population was created using purposive sampling to get various perspectives and rich data. The purposive sampling technique is a standard method to engage participants in qualitative implementation research [24]. HCPs from disciplines that were involved in the CHASE care were invited to participate in this study. HCPs were contacted via e-mail, telephone, or in person by the researcher (M.S.).

Patient selection was based on the date of their operation, the length of hospital stay, and the postoperative course (complicated or uncomplicated). As a result, patients with varying intervals between surgery and the interview, varying lengths of hospital stay, and different postoperative courses were included. They were contacted via e-mail or telephone and informed about the study. Following a period of reflection, the patients were recruited by the researcher (M.S.).

### Data collection

For this mixed-method study, qualitative data were collected through interviews with HCPs and patients, while quantitative data were collected using the MIDI questionnaire.

For the interviews with healthcare professionals, a semi-structured interview guide was developed based on the CHASE-unique elements of care (see Table 1 and Appendix 1). The interview guide for patients was developed in which all CHASE-specific steps of the pathway were evaluated in chronological order. The modified pre-, peri- and postoperative care elements were evaluated using open-ended questions regarding their perceptions of CHASE care. See Appendix 2 for the interview guide.

**Table 1:** Unique elements of the CHASE protocol and responsibilities of HCPs

Phase	CHASE element	Responsible HCP
Preoperative:	CHASE counselling	Nurse practitioner
	Admission on the day of surgery	Planner
	Preoperative analgesia with paracetamol and gabapentin	Nurse surgical ward and ward physician
	Walk to the operation theatre	Nurse surgical ward
Perioperative:	Spinal anaesthesia with hyperbaric bupivacaine (Marcaine) before induction	Anaesthesiologist
	Fluid therapy: only limited balanced crystalloids set at 3 mL/kg/h	Anaesthesiologist and anaesthesiology assistant
	Starting intra-abdominal pressure 12 mmHg, which is reduced to 8 mmHg after trocar placement	Operation nurse
	Intracorporeal primary anastomosis	Surgeon
	Specimen extraction through Pfannenstiel incision	Surgeon
Postoperative:	Analgesics with Meloxicam, Paracetamol and if necessary Oxycodone	Nurse and ward physician
	The quick stimulus of intake with an ice popsicle	Recovery room nurse
	Stop of IV-fluids Postoperative day (POD) 0–1	Nurse surgical ward
	Early mobilization on POD0	Nurse surgical ward
	Discharge on POD1 if the patient meets the following criteria: pain under control with oral analgesics (VAS < 4); no symptoms of nausea and/or vomiting; flatus or passing of stool; oral intake possible; spontaneous micturition; able to mobilize independently; no fever, tachycardia, hypotension, dyspnoea, or somnolence; confidence to go home. Patients who do not meet all the discharge criteria remain admitted until they meet all criteria	Ward physician
	Follow-up with a telephone consultation by the nurse on POD 1 and the nurse practitioner on POD 3 to evaluate recovery	Nurse practitioner

The Measurement Instrument of Determinant of Innovations (MIDI) [25] was used for quantitative analysis. The MIDI questionnaire consists of 29 determinants in four domains: the innovation (CHASE protocol), the users (HCPs), the organization (hospital) and the socio-political context (Dutch healthcare setting with accompanying laws and regulations) [25]. These determinants may positively or negatively influence the implementation. For the current study determinants that did not apply to the CHASE protocol were excluded from the questionnaire. Consequently, 13 items from 3 domains (innovation, user, and organization) were included in the questionnaire. The adapted MIDI questionnaire was discussed with two members of the research team (J.S. and S.B.); the final version is presented in Appendix 3. During the in-depth interviews with HCPs, steps that were unique to the CHASE protocol were first evaluated and subsequently, the MIDI questions were asked.

All interviews with HCPs were conducted in person by the researcher at their workplace (ZMC). Interviews with patients were conducted at one of the two ZMC hospital sites (Sittard-Geleen or Heerlen, *n* = 8) or via Microsoft Teams (*n* = 3). Before the interview, participants provided informed consent for the study and audio recording. Field notes were taken during the interview, to document participants’ facial expressions, speech, and non-verbal cues.

### Data analysis

Data collection and analysis were conducted simultaneously. All interviews were transcribed verbatim and analysed using Atlas.ti 9.0. Participant names were anonymized and replaced with numerical identifiers. The interviews and transcripts were the primary data sources for the qualitative data analysis. We analysed the data using conventional qualitative content analysis [26]. Initial codes were created based on literature findings. Codes were then modified and used to sort the interview data in a way that best summarized, integrated, and represented the content. The transcripts were read thoroughly and repeatedly to fully understand the content. Subsequently, data was coded into 6 main categories based on the results of the interview. Next, categories and subcategories were created to further specify the data proportions. The subcategories were analysed again for similarities and differences and grouped into codes. The final thematic scheme was presented as a table with all the different data categories. See Appendix 4 for the final coding template. Saturation of data was reached when no new information was extracted from the data [27]. To ensure reliability, all transcripts were reviewed by two authors independently (M.S. and E.G.). Findings were compared and conflicts were resolved when necessary. The analysis was conducted with the Dutch transcripts, the quotations were translated into English for this study; these translations underwent peer review (J.S.). The results of the MIDI questionnaires were analysed in Statistical Package for the Social Sciences (SPSS) 29.0 using descriptive analysis.

## Results

Thirteen HCPs were invited to participate, and all agreed. Twelve interviews were conducted with HCPs involved in CHASE care, including 11 individuals and one interview involving two HCPs. The median length of the interview was 25 min, ranging from 18–44 min. The participants included two anaesthesiologists, one anaesthesiology assistant, one nurse anaesthetic, one gastroenterology nurse practitioner, one surgeon, two instrumenting surgical assistants, two surgery-specialized nurses, one surgical ward physician, and two surgical planners. The population included six male and seven female HCPs. Age ranged from 28 to 65 years, with a median age of 50 years. Work experience ranged from 2 to 42 years, with a median of 20 years.

Eleven out of the 12 approached patients agreed to participate. Interviews had a median length of 40 min, ranging from 28 to 53 min. In total, five male and six female patients were interviewed. Their aged ranged from 42 to 77 years, with a median age of 72 years. Three patients had a prolonged admission (> one day), four had developed a postoperative complication. The time between surgery and interview ranged from 1 to 27 months, with a median of nine months. Baseline characteristics are displayed below.

### Interviews—HCPs

The eight categories that emerged from the evaluation of the CHASE elements were information, patient selection, CHASE protocol, execution, effect of the CHASE protocol, importance, and feedback.

#### Information

Most HCPs were supportive of the implementation of the CHASE protocol; they perceived the in-depth patient counselling and safe early discharge to be benefits of the CHASE protocol. Although the information in the protocol was clear for most HCPs, two of them suggested mentioning the rationale behind the CHASE adjustments. Protocols were printed and displayed in the operation complex by anaesthesiologists and anaesthesiology assistants.

HCP 11*: “I never have doubts or uncertainties about the protocol.”*

HCP 12: “*There is not that much background information included in the protocol…I would find that interesting.”*

#### Patient selection

Most HCPs believed that patient selection for CHASE was often adequately performed and deemed CHASE care and accelerated discharge feasible for the target population. However, one HCP questioned whether the early discharge would primarily benefit bed capacity or healthcare costs rather than patients. The majority of HCPs emphasized the critical assessment of patients’ feasibility for short stays.

HCP 7: *“Once in a while I do wonder is that a good CHASE candidate? But I don't know if you could have assessed that beforehand.”*

#### Visibility

HCPs were generally aware of patient inclusion in the CHASE protocol through notifications in the Electronic Patient Record. However, some suggested simple adjustments, such as placing a CHASE notification on patients’ beds, to improve visibility. They believed that increased visibility may result in less time spent checking patients’ CHASE status and prevent ambiguity.

HCP 4: “*CHASE is always mentioned during the TIME-OUT procedure* (safety check).*”*

HCP 5: “*Sometimes you'll really have to go looking (for a CHASE notification), but I haven't actually done that yet myself*.”

#### Protocol

Opinions about the importance of the unique CHASE steps varied, for example, the extra telephone calls were perceived as valuable by most HCPs. However, some HCPs also indicated that they did not regard all elements of the CHASE protocol as essential, e.g. there were opposing opinions on the use of spinal anaesthesia, or patients walking to the operating theatre.

HCP 7: *“…but on postoperative day three, then the patient is home for two days, I think it's nice to call then.”*

HCP 2: “…*I think you could also provide CHASE care, with discharge the day after (surgery) without spinal anaesthesia. I think that could work well in some cases too…”.*

#### Execution

Most HCPs considered the planning of CHASE patients as the second or third surgery of the day appropriate. Surgical planners reported that it was sometimes impossible to schedule CHASE patients at the desired time due to logistical or personnel difficulties. The ward nurses mentioned inconvenience with 7 AM admission at times due to the concurrent handover from the night shift. The execution of the CHASE protocol steps was considered as not complex. Some elements of CHASE were common for other patient populations, e.g. both anaesthesiologists indicated that some anaesthesia elements of the CHASE protocol were already standard practice in the ZMC or other hospitals for other surgical procedures.

HCP 5: “*For us, it is not very strenuous, or more strenuous than caring for another patient. It is getting better of course, because at the beginning, it's something you have to get used to. But because we now provide CHASE care to other patients (not colon surgery) as well, it does become more of a routine*.”

At the beginning of the protocol implementation, some steps of the CHASE protocol were not yet routinized and were forgotten, e.g. lowering intra-abdominal pressure. Colleagues helped remind the HCPs of the CHASE elements. Some HCPs indicated that they valued strict adherence to the CHASE protocol, while others used the protocol as a guideline but deviated from it depending on the patient’s condition. During busy shifts, compliance was compromised as some CHASE elements were forgotten, e.g. telephone consults. Although it was stated that the modifications in the CHASE protocol required no or little extra time, implementation could be threatened in the event of an HCP shortage.

HCP 10: *“…but I have to be honest when you have a really busy shift with two people in the late shift it sometimes gets forgotten* (telephone consult)*“.*

#### Effect

Some considered the short admission to be pleasant for patients and as complications cannot be predicted, early discharge with the discharge criteria was considered to be safe.

HCP 11: “Y*es, they (patients) do often say themselves ‘I am a CHASE patient’… ‘I can go home tomorrow’.. yes, and a CHASE patient is better prepared for that, I think, that they know that they do have to function on their own again soon.*”

#### Implementation

All HCPs indicated their commitment to providing the best possible care for patients. They considered CHASE to be an important intervention if it could improve patients’ outcomes. A few HCPs stated that CHASE should only be implemented if it is proven to be safe.

HCP 4: *"If, as a result of the CHASE protocol, a patient does indeed recover faster and experiences less pain, I think that is very important.”*

#### Feedback

HCPs reported that they lacked insight into CHASE results and received limited information about the progress of the implementation. The majority reported the desire to receive an update about progress and outcomes every 2–6 months, preferably with face-to-face conversation, presentation, or e-mail. Several HCPs stated that CHASE could also be used for other patients. To implement CHASE on a broader scale, education and training of more HCPs were considered to be important.

HCP 8*: “… it would be nice if, as being part of that cascade, we could also hear the study results or the current status of the implementation*…”.

### Results interviews – patients

The six categories that emerged from the evaluation of the CHASE elements were information, received pre-, peri, and postoperative care, discharge, and follow-up.

#### Information

In general, patients were satisfied with the method of delivery and content of the CHASE information. Face-to-face information and printed materials were appreciated, none of the patients were interested in other forms of information, e.g. a CHASE video, or additional information for the informal caregiver. Often, both the information received in the outpatient clinic as well as in the surgical ward or at the operation theatre was perceived to be straightforward. However, some patients reported the information given sometimes differed among the different HCPs they had contact with, and occasionally, information was forgotten. The majority described early discharge as the most attractive element of the CHASE protocol.

Patient 3*: “… (the information) was definitely clear. They were pretty straightforward, and I received the information folders there at the time …”.*

Patient 8: *“…there was one thing that was a bit unclear to me in the run-up to the operation, and that was whether there would be spinal anaesthesia. I got conflicting information about that (from different HCPs).”*

Patient 6: *“My motivation (for participation) was that I was told that the recovery was very fast.”*

#### Execution—Preoperative care

Most of the specific CHASE care was perceived as positive, e.g. walking to the operation theatre. Patients reported no side effects of the preoperative medication. Admission on the morning of the surgery went well, some patients had to wait longer for intake with the nurse.

Patient 1: *“I liked the fact that I walked to the operating theatre…yes, because if you are in that bed you are stigmatized as a patient anyway, and of course, I can walk to the operating theatre …”.*

#### Execution—Perioperative care

Nine patients reported that they had little to no inconvenience by the spinal anaesthesia. On the other hand, two patients reported that the injection of spinal anaesthesia and the effect of the anaesthesia wearing off were unpleasant.

Patient 3: *“So, I felt in my whole body something was not right, because the spinal anaesthesia was wearing off which I felt in my legs. They started to tingle again, and my head became so light, I was really light-headed due to the anaesthesia. So, I experienced that as an unpleasant feeling.”*

#### Execution—Postoperative care

The patients felt encouraged by the nursing staff to start mobilization and intake early on. All patients reported receiving adequate guidance from the nurse on mobilisation. Removal of the bladder catheter was desired before 10 AM.

Patient 2:* “It must have been six, six-thirty and then I was given a cup of tea and a dry biscuit, I think…then a nurse came, who intensively mobilized with me and walked with me from the bed to the toilet, yes, she did that very well.”*

Postoperative pain was well controlled for most patients. Of the three patients admitted for longer than one day, one developed an anastomotic leakage. The two other patients remained admitted due to postoperative nausea and vomiting and uncertainty about going home. Patients who developed a complication reported they did not feel that this complication was causally related to the CHASE protocol.

Patient 8: *“No, I don't think I got sick because I went home too early. No, that anastomotic leakage is not from going home I think.”*

#### Discharge

Discharge criteria were understandable for all patients, and most agreed with the decision made to be discharged. If patients did not feel ready to be discharged, they felt the opportunity to discuss the time of discharge with the ward physician.

Patient 2*: “The surgeon who operated on me and the ward physician came together”. Interviewer: “They came to see how you were doing.” Patient: “Yes, 'how is it going?', ‘Yes, fine’, ‘Good, well then you can go home’. Fine, I also wanted to leave… I had also set myself up for that”.*

Patients received all essential information before discharge by the nurses, this was regarded as clear. The paper discharge leaflet was considered to be sufficient.

Patient 9: *“She also said ‘If you have any complaints, or if is there anything, or you have any questions, always call’. I was given two numbers for daytime and evening.”*

#### Execution—Follow-up

Telephonic aftercare was not received by all patients. Those who received it (*n* = 7) all highly appreciated it. It was experienced as proximity and personalization of care. Patients unanimously reported that they felt safe recovering at home.

Patient 4: “*I thought it would be fine because they don't let you go home anyway if something is not right, so I trusted that, and I had that confidence from the start.”*

Patient 6:* “Yes I liked it, that part (telephone consult) of aftercare is nice…it gives you the feeling that you are not a number and that, yes, you do matter.”*

### Results MIDI-questionnaire

**Fig. 1:**
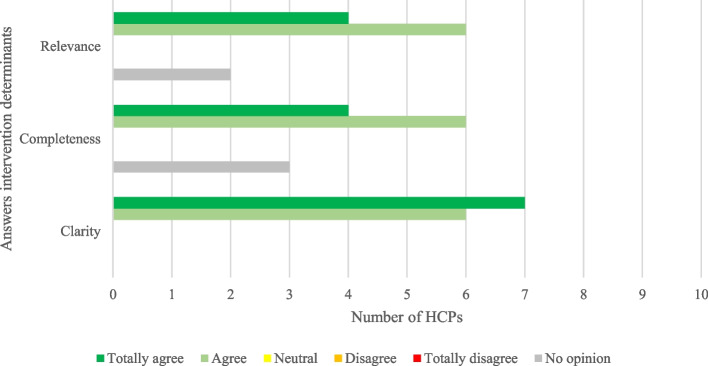
Answers on CHASE intervention determinants

Overall, HCPs were positive about the CHASE intervention; answers to the MIDI questionnaire items were mostly neutral or positive about the intervention. The HCP identified several facilitators and barriers that will be further discussed. We report the responses given by the HCPs. Regarding determinants of the intervention, one of the most important facilitators was the relevance of the CHASE study (see Fig. 1). The majority of HCPs considered the CHASE protocol to be comprehensible and complete. Three HCPs intentionally did not score the completeness as they only focused on part of the protocol relevant to their work and, therefore, felt they could not assess completeness for other specialities.

**Fig. 2:**
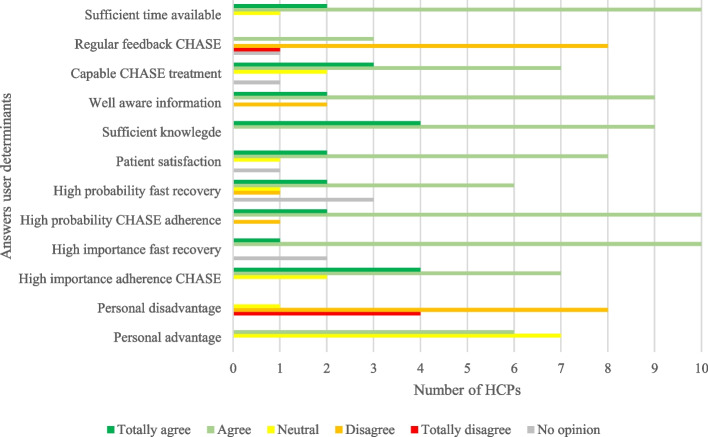
Answers on CHASE user determinants

The importance of adherence to CHASE and accelerated recovery, high probability of adherence to CHASE, high patient satisfaction and a sense of good education were valuable facilitators (see Fig. 2).

The main barrier was the absence of regular feedback regarding the CHASE implementation, almost all HCPs reported not having received updates on the implementation progress or results.

**Fig. 3:**
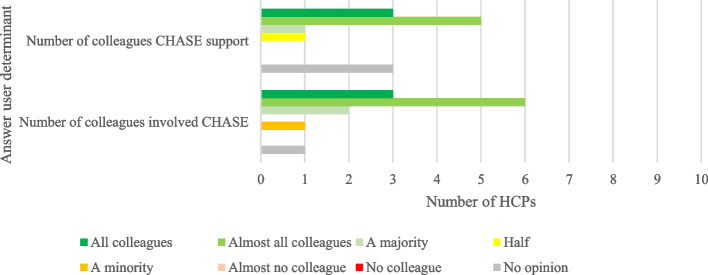
Answers on CHASE user determinants—colleagues

**Fig. 4:**
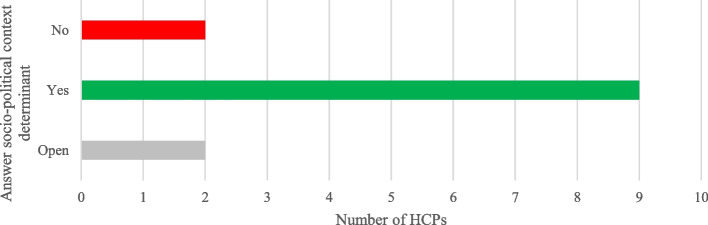
Answers on socio-politic context determinants – presence coordinator

The number of colleagues participating in CHASE was lower than the number of colleagues who could provide support, most often a CHASE coordinator was present (see Figs. 3 and 4).

## Discussion

While a growing number of studies demonstrate the feasibility and safety of accelerated ERPs after colon surgery, only a few studies have been conducted to assess HCPs’ and patients’ perspectives. The purpose of this study was to describe the perspectives of HCPs and patients on an accelerated ERP (CHASE) to improve the implementation process. This study identified high satisfaction of both HCPs and patients with the CHASE accelerated ERP. HCPs and patients positively reviewed education. The majority of HCPs deemed the CHASE protocol to be a relevant innovation and found themselves aware and capable of providing CHASE care. A minority of HCPs and patients considered specific CHASE elements to be less convenient, e.g. spinal anaesthesia. This study emphasises the benefits of profound education for both HCPs and patients and the negative effect of lack of regular feedback. Barriers also included a lack of confidence in the added value of certain CHASE elements and an insufficient number of involved HCPs.

Due to a limited number of studies describing the views of stakeholders with accelerated ERPs, comparing results to literature is challenging [28]. Since CHASE entails optimized ERAS elements, results are linked to ERAS studies as well.

### HCPs

First, consistent with the literature, the HCPs considered a clear and concise multidisciplinary protocol to be an important facilitator for implementing complex interventions [29]. This finding corroborates the findings of previous studies that having a standardized guideline facilitates protocol implementation [15, 17, 18, 30].

Education about the CHASE intervention for HCPs consisted of protocols and oral information provided by the CHASE research team. This information was perceived to be sufficient. However, during interviews, patients stressed the importance of training/education as inconsistent information between HCPs was considered to be an important barrier to participation. This suggests that not all HCPs were, in fact, adequately educated or that the patients misperceived the provided information. The importance of thorough staff education is strongly supported by previous studies [17, 30, 31]. For the education of complex interventions such as ERAS, these studies suggest i.e. small-scale educational booster meetings, reminders and network meetings. Expert consensus also supported including modules or e-learning in training programs [32].

The HCPs considered adherence to the protocol to be important, nonetheless not all of them recounted the likelihood of high adherence to be high. During the interviews, some HCPs mentioned that full compliance with the protocol was difficult. The reasons included a lack of time, the fact that the CHASE protocol was not yet a standard routine or the need for adjustments to provide patient-centred care. This result is supported by the findings of other implementation studies [15, 32, 33]. Prior studies demonstrated that high levels of adherence are essential to achieve the intended benefits for patients [4, 34]. Regular feedback and monitoring are often mentioned as important facilitators of implementation, as in this study. Recommendations to improve compliance included regular feedback on patient outcomes and progress of implementation; this was underlined by a reported desire for feedback by the majority of HCPs [15, 17, 18, 30]. Apart from regular feedback, setting targets or appointing an HCP as a ‘champion’ to enthusiastically promote and facilitate the implementation of innovation may improve implementation [35, 36]. On the other hand, the field of tension between strict protocol adherence and patient-centred care remains. Some HCPs stated they deliberately deviated from the protocol if a patient’s condition required personalized care. This could be addressed by including flexible components in the CHASE protocol [15].

An increase in the visibility of the CHASE patients was suggested to facilitate adherence, this is also supported by the previous studies [15, 37].

Patient-related factors that facilitated CHASE care were the ability to see the impact of the CHASE care provided. For instance, patient’s eagerness to go home on postoperative day one, adequate pain control and positive feedback, which accords with the earlier findings of Levy et al. [38]. Some HCPs mentioned this to be an important motivator to maintain and adhere to the CHASE protocol. Also, HCPs noticed that quick turnover of patients attributed to the increase in hospital bed availability.

The modifications made in the CHASE protocol were considered easy to implement, which enabled its implementation; this corroborates the earlier findings of Gotlib Conn et al. [37]. Some elements of the CHASE protocol, such as low intraabdominal pressure, were also adopted for other surgical interventions. Similar to ERAS, accelerated ERPs such as CHASE could plausibly be implemented within the surgical field [39].

The CHASE research team included HCPs from all different specialities involved in the broad CHASE care. The research team was also the link between the CHASE intervention and the HCPs providing CHASE care. Most HCPs referred to the CHASE team member of their speciality as the CHASE coordinator within their specialism, who informed and updated them about the CHASE protocol and progress. HCPs that lacked a coordinator considered this to be a necessity. This supports the evidence from previous studies in which leadership was demonstrated to be important [30, 31].

### Patients

The high patient satisfaction in this study aligns with the work of Curfman et al., who demonstrated a pleasant patient experience with an accelerated ERP [28]. Both patients with an uncomplicated postoperative course and those with a complicated postoperative course felt safe during hospital and at-home recovery. Most often, patients agreed with the ward physician’s discharge evaluation. In case of disagreements, patients felt that there were ample opportunities to discuss any concerns with the physician and prolong hospital admission. This is consistent with the findings of previous studies describing the importance of maintaining the option to personalize elements of care [28, 40].

With the accelerated ERP CHASE being in its early stages, the optimal protocol remains undetermined. The CHASE protocol was shown to facilitate discharge on POD1 for 80% of the study population [8]. The adjusted pain management consisted of spinal anaesthesia. Few patients reported discomfort with the injection of the anaesthetic agent or the wearing-off effect. Patients with an uncomplicated postoperative course reported little to no postoperative pain. Pain scores were lower than reported by Levy et al., this can be explained by the fact they described pain scores during movement and coughing only [38].

It has been reported that patient education and understanding of discharge criteria are of vital importance to enhanced recovery [28, 40, 41]. The optimal method for patient education has not been determined yet [42]. Overall, patients expressed satisfaction with the information they received and for the vast majority, discharge criteria were clear. Some reported their desire to get more information about the expected course of recovery at home. They felt that giving all this information preoperatively would be overwhelming but would have appreciated a more extensive printed or digital information form.

Future studies of the CHASE protocol should focus on assessing the effectiveness of the implementation strategies. Also, studies determining the best method for patient education are needed. Once other hospitals have also implemented accelerated enhanced recovery, their perspectives could be assessed to increase the generalisability of the studies.

#### Strengths/weaknesses

The strength of this study is that, to our knowledge, it is the first study to explore the perspectives of both HCPs and patients on the implementation of an accelerated ERP programme. Research that identifies factors that potentially influence the implementation in different settings is important to help advance and promote improvement [43]. Another strength is the broad range of stakeholders included in the study, encompassing patients, allied HCPs and managers. This is essential because this accelerated ERP, similar to ERAS, is a complex intervention requiring support from several stakeholders [44]. The mixed-method design allowed the combination of qualitative data about the perspectives of HCPs and patients with quantitative data about implementation determinants reviewed by HCPs using the validated MIDI questionnaire [45].

The findings of this study can be limited by the fact that it is a retrospective review of patient experiences. The time between surgery and the interview ranged from one month to 2 years. Patients’ memory may have faded, especially for those who underwent surgery in the early stages of CHASE implementation. There may also be recall bias, and the course of recovery might have influenced the review of their experience with CHASE. As a single-centre study; these findings cannot be extrapolated to all patients. Although purposive sampling was conducted, caution must be applied due to the small sample size, and the results might not be fully representative of all patients and HCPs involved in CHASE or other accelerated ERPs. The studies were conducted by an MD, currently working as a surgical Ph.D. student. Since the student has regular contact with the HCPs, their answers may have been affected by this relationship, as well as by the Ph.D. student’s involvement in the CHASE study. On the other hand, the relationship is not a direct working relationship, and the Ph.D. student was not the initiator of the CHASE protocol. Additionally, there is a possibility of attribution bias based on the subjective interpretation of the researchers involved in coding (M.S. and E.G.) regarding the answers and statements given by the participants. Since one of the researchers (E.G.) was not involved in the CHASE study, the bias is considered to be limited.

## Conclusion

This is the first study to describe the patients’ and HCPs’ perspectives on an accelerated ERP for elective colon cancer surgery. The vast majority of participants were positive about the innovation of this 23-h enhanced recovery pathway, CHASE. The combination of standardizing care, profound patient education, the opportunity to safely discharge patients earlier, and the positively reviewed aftercare innovation increased adherence to CHASE care.

### Supplementary Information

**Supplementary Material 1.**

**Supplementary Material 2.**

**Supplementary Material 3.**

**Supplementary Material 4.**

## Data Availability

The datasets used and analysed during the current study are available from the corresponding author on reasonable request.
