# Supplementary figures and images for: Views and experiences of healthcare professionals and patients on the implementation of a 23-hour accelerated enhanced recovery programme: a mixed-method study
Source: BMC Health Serv Res. 2024 Mar 13;24:330. doi: 10.1186/s12913-024-10837-z (PMC10935952; doi:10.1186/s12913-024-10837-z)

Appendix 1 - Flowchart CHASE care

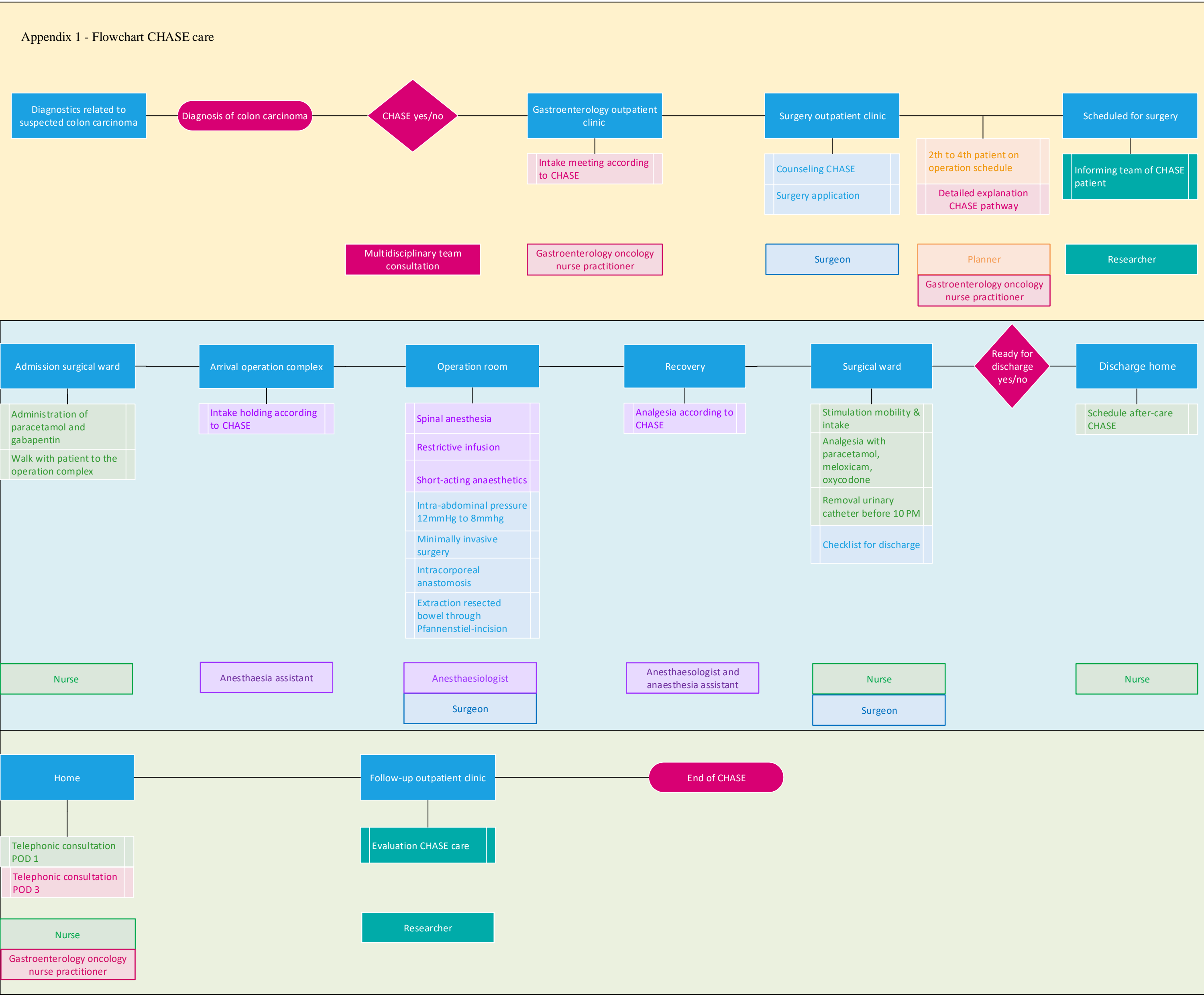

Supplement: Supplementary file 1 — Supplementary Material 1. [file 12913_2024_10837_MOESM1_ESM.pdf]
