# Supplementary material for: Views and experiences of healthcare professionals and patients on the implementation of a 23-hour accelerated enhanced recovery programme: a mixed-method study
Source: BMC Health Serv Res. 2024 Mar 13;24:330. doi: 10.1186/s12913-024-10837-z (PMC10935952; doi:10.1186/s12913-024-10837-z)
Supplement: Supplementary file 2 — Supplementary Material 2. [file 12913_2024_10837_MOESM2_ESM.docx]

Appendix 2 – Interview guide patients

**A. Roadmap (extended version)**

1. **Introduction interview** (no audio recording)

Welcome! (introducing yourself + offering coffee/tea)

The interview to which I have invited you is about the accelerated recovery programme you participated in here at Zuyderland in 2020 and 2021. This programme took place just before, during and after your surgery to accelerate your recovery after surgery. The accelerated recovery programme consisted of adjustments in pain relief, fluid administration and full-surgery via keyhole surgery, among other things. The aim of the study at the time was to see if accelerated recovery would be feasible and successful. For 80% of the patients, it turned out to be feasible to go home after one day of admission to further recover. We will implement the protocol in practice and make it accessible to as many patients as possible. By improving the accessibility, we mean adjusting the protocol where necessary to make accelerated recovery as pleasant and successful as possible. Since you are the expert by experience in this field, we are curious to hear about your experiences with the accelerated recovery programme.

Let me stress again that we guarantee anonymity and confidential treatment of your data. The interview will be recorded, if you agree, so that we can analyse it as best we can afterwards. Only I and my supervisor are aware of your data, otherwise we keep it strictly anonymous. The results of the interviews will therefore be processed and presented anonymously. The interview will last half an hour to a maximum of an hour. In it, I will go through all parts of the accelerated recovery programme with you step by step. Do you have any questions beforehand?

To keep the thread of the interview and to make sure that all parts are covered, I will also take short notes during the interview.

1. **Topic list** (chronological):
   1. *Providing information on the accelerated recovery programme outpatient clinic*

Opening question: What did you think of the information on the CHASE programme?

- - 1. From whom did you get the information?
    2. Adequacy of information (clear/motivational/etc.)
    3. Thoughts/feelings/considerations on the information

What went well? What could be better? How can it be improved?

- 1. *Motivation for participation*

Opening question: What was your motivation for joining the accelerated recovery programme?

What factors played into this decision?

- 1. *Component: hospitalisation*

Opening question: How did you find the admission for the CHASE recovery programme?

- - 1. Information about the course of CHASE during admission
    2. Experience with analgesics (paracetamol and gabapentin), e.g. side effects
    3. Experience of walking to the surgery complex

What went well? What could be better? How can it be improved?

- 1. *Component: holding*

Opening question: How did you find admission on the holding?

- - 1. How was the education on the course of CHASE anaesthesia?

What went well? What could be better? How could it be better? How could it be better?

- 1. *Component: operation*

Opening question: With what feeling did you go into the surgery? Different by CHASE programme?

- - 1. How did you experience the spinal anaesthesia?

What went well? What could be better? How can it be improved?

- 1. *Component: recovery*
     1. Were you in pain when you woke up from anaesthesia?

What went well? What could be better? How can it be improved?

- 1. *Component: surgical ward*

Opening question: How did you experience your postoperative stay in the nursing ward regarding accelerated recovery programme?

- - 1. Contact nurses and ward physician
    2. Stimulus to mobilise?
    3. Stimulus to start intake?
    4. Experience of pain and analgesia

What went well? What could be better? How can it be improved?

- 1. *Component: discharge*
     1. Information on discharge criteria accelerated recovery programme
     2. Information on course of discharge in accelerated recovery programme
     3. Explanation of course of postoperative recovery CHASE

What went well? What could be better? How can it be improved?

- 1. *Component: aftercare*

Opening question: How did you experience the CHASE aftercare?

- - 1. Experience regarding telephone consultation nurse and nurse specialist
    2. Experience regarding evaluation CHASE care

What went well? What could be better? How can it be improved?

- 1. Component: recovery at home
     1. How did you feel about going home after one day?
     2. How did recovery go at home?

What went well? What could be better? How can it be improved?

1. **Closing interview session** (stop audio recorder)

I have come to the end of the interview. Did you miss anything, or do you have anything to add?

What did you think of the interview? How did you experience the interview? Do you have any tips and tops for me as an interviewer?

Are you interested in receiving the final result?
