# Supplementary material for: Views and experiences of healthcare professionals and patients on the implementation of a 23-hour accelerated enhanced recovery programme: a mixed-method study
Source: BMC Health Serv Res. 2024 Mar 13;24:330. doi: 10.1186/s12913-024-10837-z (PMC10935952; doi:10.1186/s12913-024-10837-z)
Supplement: Supplementary file 3 — Supplementary Material 3. [file 12913_2024_10837_MOESM3_ESM.docx]

Appendix 3 – MIDI-Questionnaire

| **Determinants related to the innovation** | Fully disagree | Disagree | Neutral | Agree | Fully agree |
| --- | --- | --- | --- | --- | --- |
| *Procedural clarity*  The CHASE protocol is described in clear steps. It is clear which actions to perform in which order. |  |  |  |  |  |
| *Completeness*  The CHASE protocol is complete; all necessary information is included in the protocol |  |  |  |  |  |
| *Relevance target population*  I think the CHASE protocol is a relevant innovation for the target population (ASA I-II patients undergoing elective, oncological colon resection) |  |  |  |  |  |

| **Determinants related to the user** | Fully disagree | Disagree | Neutral | Agree | Fully agree |
| --- | --- | --- | --- | --- | --- |
| *Personal advantage/disadvantage*  The CHASE protocol has a positive impact on my way of working |  |  |  |  |  |
| Please specify the advantages | | | | | |
| The CHASE protocol has a negative impact on my way of working |  |  |  |  |  |
| Please specify the disadvantages | | | | | |
| *Outcome expectations*  I find it important that |  |  |  |  |  |
| - the CHASE patient is treated according to protocol |  |  |  |  |  |
| - the CHASE patient recovers fast |  |  |  |  |  |
| I expect that |  |  |  |  |  |
| - the CHASE patient is treated according to protocol |  |  |  |  |  |
| - the CHASE patient recovers fast |  |  |  |  |  |
| *Satisfaction client*  I expect the patient to be satisfied with the CHASE protocol |  |  |  |  |  |
| *Knowledge (subjective)*  I have sufficient knowledge to perform the CHASE protocol |  |  |  |  |  |
| *Information processing*  I know where to find information about the CHASE protocol |  |  |  |  |  |
| *Own-effectiveness expectation*  I can successfully perform the required actions of the CHASE protocol |  |  |  |  |  |

| *Descriptive norm* | None of my colleagues | Almost none of my colleagues | A minority | Half of my colleagues | A majority | Almost all of my colleagues | All colleagues |
| --- | --- | --- | --- | --- | --- | --- | --- |
| How many of your colleagues provide protocol-based care to CHASE protocol-eligible patients? |  |  |  |  |  |  |  |
| I can count on sufficient support from my colleagues in case I need it when using the CHASE protocol |  |  |  |  |  |  |  |

| **Determinants related to the surrounding** | Fully disagree | Disagree | Neutral | Agree | Fully agree |
| --- | --- | --- | --- | --- | --- |
| *Feedback to the user*  There is regular feedback on the progress of the implementation of the CHASE protocol |  |  |  |  |  |
| *Time*  Our organisation provides me with sufficient time to integrate the CHASE protocol into my daily work |  |  |  |  |  |

*Coordinator*

**Within my field, one or more colleagues have been assigned to coordinate the implementation of the CHASE protocol:**

- Yes
- No
