# Supplementary material for: Views and experiences of healthcare professionals and patients on the implementation of a 23-hour accelerated enhanced recovery programme: a mixed-method study
Source: BMC Health Serv Res. 2024 Mar 13;24:330. doi: 10.1186/s12913-024-10837-z (PMC10935952; doi:10.1186/s12913-024-10837-z)
Supplement: Supplementary file 4 — Supplementary Material 4. [file 12913_2024_10837_MOESM4_ESM.docx]

Appendix 4 – Coding template

| Main Category | Category | Subcategory | Code |
| --- | --- | --- | --- |
| A Information CHASE care | **A.1** Way of delivery | **A.1.1** Provider | **A.1.1** Provider |
|  |  | **A.1.2** Manner | **A.2.1** Printed |
|  |  |  | **A.2.2** Digital |
|  |  |  | **A.2.3** Oral |
|  | **A.2** Content |  | **A.1.3** Accessibility |
|  |  |  | **A.2.1** Completeness |
|  |  |  | **A.2.2** Comprehensiveness |
|  | **A.3** Perception |  | **A.3.1.** Motivation participation |
|  |  |  | **A.3.2.** Adjustments |
|  |  |  | **A.3.3.** Knowledge protocol |
| B. Application CHASE | **B.1** Patient selection | **B.1.1.** Adequacy of patient selection | **B.1.1.1** Adequate |
|  |  |  | **B.1.1.2.** Inadequate |
|  | **B.2** Recognition CHASE patients |  | **B.2.1** Manner |
|  |  |  | **B.2.2** Timing |
|  |  |  | **B.2.3** (Ways of) improvement |
|  | **B.3** Other study population |  | **B.3.1** Other potential population |
| C. Protocol | **C.1** Elements CHASE care |  | **C.1.1** Beneficial elements |
|  |  |  | **C.1.2** Detrimental elements |
| D. Execution | **D.1** Preoperative care | **D.1.1** Diagnostics | **D.1.1.1** Gastroenterology |
|  |  |  | **D.1.1.2** Other |
|  |  | **D.1.2** Screening | **D.1.2.1** Preoperative screening |
|  |  | **D.1.3** Planning | **D.1.3.1** Time of surgery |
|  |  |  | **D.1.3.2** Barriers |
|  |  |  | **D.1.3.3** Facilitators |
|  |  |  | **D.1.3.4** Importance |
|  | **D.2** Perioperative CHASE care | **D.2.1** Surgical ward | **D.2.1.1** Admission |
|  |  |  | **D.2.1.2** Preoperative care |
|  |  |  | **D.2.1.3** Walking to the operation theatre |
|  |  | **D.2.2** Holding | **D.2.2.1** Preparation |
|  |  |  | **D.2.2.2** Intake holding |
|  |  | **D.2.3** Operating room | **D.2.3.1** Time-Out |
|  |  |  | **D.2.3.2** Spinal anaesthesia |
|  |  |  | **D.2.3.3** Urinary catheter |
|  |  |  | **D.2.3.4** Intra-abdominal pressure |
|  |  |  | **D.2.3.5** Other |
|  | **D.3** Postoperative care | **D.3.1** Recovery ward | **D.3.1.1** Recovery ward - general |
|  |  | **D.3.2** Surgical ward | **D.3.2.1** Mobilization |
|  |  |  | **D.3.2.2** Urinary Catheter |
|  |  |  | **D.3.2.3** Medication |
|  |  |  | **D.3.2.4** Discharge instructions |
|  |  |  | **D.3.2.5** Postoperative telephone consult |
|  |  |  | **D.3.2.6** General |
|  |  | **D.3.3** Surgeon / surgical resident | **D.3.3.1** Discharge assessment |
|  |  |  | **D.3.3.2** Outpatient clinic |
|  |  | **D.3.4** General | **D.3.4.1** General |
|  | **D.4.** (Home) recovery | **D.4.1** General | **D.4.1.1** General |
|  |  |  | **D.4.1.2** Pain |
|  |  |  | **D.4.1.3** Mobilization |
|  |  |  | **D.4.1.4** Intake |
|  |  | **D.4.2** Care | **D.4.2.1** Medical care |
|  |  |  | **D.4.2.2** Informal care |
|  |  |  | **D.4.2.3** Follow-up |
|  |  | **D.4.3** Postoperative recovery | **D.4.3.1** Uncomplicated |
|  |  |  | **D.4.3.2** Complicated |
| E. Effect |  |  | **E.1** General |
|  |  |  | **E.2** Impact |
|  |  |  | **E.3** Way of working |
| F. Implementation |  |  | **F.1** Importance |
|  |  |  | **F.2** Time |
|  |  |  | **F.3** Needs |
|  |  |  | **F.4** Communication |
|  |  |  | **F.5** Population |
|  |  |  | **F.6** Team |
| G. Feedback |  |  | **G.1** Results |
|  |  |  | **G.2** Content |
|  |  |  | **G.3** Timing/frequency |
|  |  |  | **G.4** Mode |
|  |  |  | **G.5** Audience |
